# Safety of Percutaneous Endoscopic Gastrostomy Placement in Pregnancy: A Case Report and Literature Review

**DOI:** 10.1155/2022/2599274

**Published:** 2022-01-08

**Authors:** Celine Aslinia, Armand Edalati, Arianna Fallahian, Arya Edalati, Maha Hosseini

**Affiliations:** ^1^Indiana University School of Medicine, Indianapolis, IN, USA; ^2^University of Missouri, Kansas City, MO, USA; ^3^University of Kansas Medical Center, Kansas City, KS, USA

## Abstract

Gastrostomy tube placement in pregnancy is historically contraindicated due to risk of injury to the developing fetus and exposure to anesthetic agents. However, in cases where oral nutritional access is severely jeopardized, percutaneous endoscopic gastrostomy (PEG) tube placement can be a life-saving measure. In this case report and literature review, we present a case of successful PEG placement in a pregnant woman, followed by a discussion of the existing literature regarding PEG placement during pregnancy.

## 1. Case Presentation

A 24-year-old woman was found down in cardiac arrest, hypoxic with agonal breathing. Field intubation was performed by paramedics. The suspected etiology for cardiac arrest was thought to be unintentional illicit drug overdose. She was diagnosed with irreversible hypoxic brain injury resulting in a permanent vegetative state. During emergency room assessment, she was found to be pregnant, with the estimated age of pregnancy as the second trimester. Five days later, she underwent a tracheostomy and a gastroenterology consult was placed for a PEG tube. Abdominal examination revealed a gravid uterus above the umbilicus. After discussing the risks, benefits, and alternatives to feeding tube placement with the patient's power of attorney, a 24 Fr externally removable gastrostomy tube was placed endoscopically using the pull technique. Estimated blood loss was minimal, and the patient tolerated the procedure well. There was no evidence of damage or injury to the gravid uterus. This patient was under close surveillance by the obstetrics group managing her pregnancy as she stayed in the hospital for the remainder of her pregnancy due to her tracheostomy and gastrostomy status as well as her vegetative state. Physical examination and obstetric examination after PEG placement did not reveal any of the following, which could have represented a complication of PEG placement: ruptured membrane, preterm labor, vaginal bleeding, chorioamnionitis, or miscarriage. She successfully completed the course of pregnancy and went into spontaneous labor at week 38. Due to the transverse positioning of the fetus, initial delivery with forceps was unsuccessful and emergency C-section was performed. A healthy, term baby was born with an Apgar score of 8/9 out of 10 [1]. The mother was discharged under hospice care after delivery.

## 2. Discussion

Historically, performing procedures that require sedation and instrumentation of the abdominal cavity is not advised during pregnancy. However, there are circumstances where surgery or endoscopy is inevitable. We presented a case of successful PEG placement during the second trimester of pregnancy without compromising the pregnancy outcome.

Other cases in the literature have reported patients with PEG tube placement as early as 8 weeks of gestation [2] and as late as 29 weeks of gestation [3], spanning a range that covers all three trimesters. Nasogastric (NG) tube placement, while less invasive, is a much less sustainable option over a longer period due to the risks of nasal septal necrosis [4], chronic sinusitis [5], or accidental or intentional removal by the patient [6]. Laparoscopic gastrostomy tube placement is a technically acceptable approach in the second trimester of pregnancy [7]. However, as our center had the expertise to perform endoscopic placement of gastrostomy tube, which only requires moderate sedation or monitored anesthesia care without general anesthesia, the decision was made to proceed with percutaneous endoscopic gastrostomy to limit fetal exposure to anesthetic agents in this case. If the gestational age was in the third trimester of pregnancy, laparoscopy could have been a safer option due to a large, gravid uterus.

PEG tube placements in pregnant patients have most commonly been due to impaired nutritional status because of hyperemesis gravidarum [3, 8–12] or anorexia [6], as well as traumatic brain injury through motor vehicle accidents [13] or intraparenchymal hemorrhage [14]. To date, there have been no published cases regarding hypoxic brain injury or suspected drug overdose in a pregnant person necessitating PEG tube placement.

Senadhi et al. described multiple reasons for the hesitance to perform PEG placement in the pregnant population, especially in regards to the potential to induce premature labor, damage fetal structures, or result in infection. However, our case as well as review of the existing data revealed no adverse effect on pregnancy outcome after PEG placement, indicating the safety of this intervention in the setting of compromised nutrition status in pregnant women.

## Data Availability

No data were used to support this study.

## References

[1] Simon L. V., Hashmi M. F., Bragg B. N. (2021). APGAR score. *StatPearls. Treasure Island (FL)*.

[2] Wejda B., Soennichsen B., Huchzermeyer H., Mayr B., Cirkel U., Dormann A. J. (2003). Successful jejunal nutrition therapy in a pregnant patient with apallic syndrome. *Clinical Nutrition*.

[3] Godil A., Chen Y. K. (1998). Percutaneous endoscopic gastrostomy for nutrition support in pregnancy associated with hyperemesis gravidarum and anorexia nervosa. *Journal of Parenteral and Enteral Nutrition*.

[4] Lai P. B., Pang P. C., Chan S. K., Lau W. Y. (2001). Necrosis of the nasal ala after improper taping of a nasogastric tube. *International Journal of Clinical Practice*.

[5] Adeyemo A. A., Fasunla A. J., Adeosun A. A., Abdullahi H. (2007). Rhinosinusitis; a potential hazard of nasogastric tube insertion. *Annals of Ibadan Postgraduate Medicine*.

[6] Shaheen N. J., Crosby M. A., Grimm I. S., Isaacs K. (1997). The use of percutaneous endoscopic gastrostomy in pregnancy. *Gastrointestinal Endoscopy*.

[7] Oelsner G., Stockheim D., Soriano D. (2003). Pregnancy outcome after laparoscopy or laparotomy in pregnancy. *Journal of the American Association of Gynecologic Laparoscopists*.

[8] Garg S., Contag S., Dutta S. (2014). Emerging role of endoscopically placed jejunostomy tubes in the management of severe hyperemesis gravidarum: a case series. *Gastrointestinal Endoscopy*.

[9] Irving P. M., Howell R. J., Shidrawi R. G. (2004). Percutaneous endoscopic gastrostomy with a jejunal port for severe hyperemesis gravidarum. *European Journal of Gastroenterology and Hepatology*.

[10] Kruchko D., Shah N., Broy C., Silas D. (2020). Percutaneous endoscopic jejunostomy tube placement for treatment of severe hyperemesis gravidarum in pregnancy. *Journal of Investigative Medicine High Impact Case Reports*.

[11] Rupani S. V., Ergen W. F., Weber F., Peter S. (2018). Direct percutaneous endoscopic jejunostomy for the management of gastroparesis in pregnancy. *Obstetrics & Gynecology*.

[12] Saha S., Loranger D., Pricolo V., Degli-Esposti S. (2009). Feeding jejunostomy for the treatment of severe hyperemesis gravidarum: a case series. *Journal of Parenteral and Enteral Nutrition*.

[13] Koh M. L., Lipkin E. W. (1993). Nutrition support of a pregnant comatose patient via percutaneous endoscopic gastrostomy. *Journal of Parenteral and Enteral Nutrition*.

[14] Senadhi V., Chaudhary J., Dutta S. (2010). Percutaneous endoscopic gastrostomy placement during pregnancy in the critical care setting. *Endoscopy*.

